# High Genetic Addiction Risk Score (GARS) in Chronically Prescribed Severe Chronic Opioid Probands Attending Multi-pain Clinics: an Open Clinical Pilot Trial

**DOI:** 10.1007/s12035-021-02312-1

**Published:** 2021-03-08

**Authors:** Mark Moran, Kenneth Blum, Jessica Valdez Ponce, Lisa Lott, Marjorie C. Gondré–Lewis, Sampada Badgaiyan, Raymond Brewer, B. William Downs, Philip Fynman, Alexander Weingarten, Jean Lud Cadet, David E. Smith, David Baron, Panayotis K. Thanos, Edward J. Modestino, Rajendra D. Badgaiyan, Igor Elman, Mark S. Gold

**Affiliations:** 1 Pain Consultants of San Antonio, San Antonio, TX USA; 2 Department of Nutrigenomics, Geneus Health, LLC, San Antonio, TX USA; 3 Department of Psychiatry, University of Vermont, Burlington, VT USA; 4 Division of Addiction Services, Dominion Diagnostics, North Kingston, RI USA; 5 Western University Health Science Centers, Graduate College, Pompano, CA USA; 6 Institute of Psychology, Eotvos Loránd University, Budapest, Hungary; 7 Department of Psychiatry, Wright University Boonshoft School of Medicine, Dayton, OH USA; 8 The Kenneth Blum Behavioral and Neurogenetic Institute, (Division of Ivitalize Inc), Austin, TX USA; 9 Division of Nutrigenomics, Victory Nutrition International, LLC, Lederoch, PA USA; 10 Division of Clinical Neurology, Path Foundation NY, New York, NY USA; 11 Department of Anatomy, Developmental Neuro-Psycho-Pharmacology Laboratory, Howard University College of Medicine, Washington, DC USA; 12 Comprehensive Pain Management, Syosset, NY USA; 13 Department of Psychiatry, New York Medical College, Valhalla, NY USA; 14 Molecular Neuropsychiatry Branch, National Institutes of Health, Bethesda, MD USA; 15 Department of Pharmacology, College of Medicine, University of San Francisco, San Francisco, CA USA; 16 Department of Psychology & Behavioral Neuropharmacology and Neuroimaging Laboratory on Addictions (BNNLA), Research Institute on Addictions, University at Buffalo, Buffalo, NY USA; 17 Department of Psychology, Curry College, Milton, MA USA; 18 Department of Psychiatry, Icahn School of Medicine Mt Sinai, New York, NY USA; 19 Department of Psychiatry, South Texas Veteran Health Care System, Audie L. Murphy Memorial VA Hospital, San Antonio, TX USA; 20 Long School of Medicine, University of Texas Medical Center, San Antonio, TX USA; 21 Center For Psychiatric Medicine, Lawrence, MA USA; 22 Department of Psychiatry, Harvard School of Medicine, Boston, MA USA; 23 Department of Psychiatry, Harvard University School of Medicine, Cambridge, MA USA; 24 Department of Psychiatry, Washington University School of Medicine, St. Louis, MO USA

**Keywords:** Pain, Opioids, GARS, Hyperalgesia, Tolerance, Hypodopaminergia, Polymorphisms, Neuroadaptation, Genetic risk

## Abstract

Millions of Americans experience pain daily. In 2017, opioid overdose claimed 64,000 lives increasing to 84,000 lives in 2020, resulting in a decrease in national life expectancy. Chronic opioid use results in dependency, drug tolerance, neuroadaptation, hyperalgesia, potential addictive behaviors, or Reward Deficiency Syndrome (RDS) caused by a hypodopaminergia. Evaluation of pain clinic patients with the Genetic Addiction Risk Score (GARS) test and the Addiction Severity Index (ASI- Media Version V) revealed that GARS scores equal to or greater than 4 and 7 alleles significantly predicted drug and alcohol severity, respectively. We utilized RT-PCR for SNP genotyping and multiplex PCR/capillary electrophoresis for fragment analysis of the role of eleven alleles in a ten-reward gene panel, reflecting the activity of brain reward circuitry in 121 chronic opioid users. The study consisted of 55 males and 66 females averaging ages 54 and 53 years of age, respectively. The patients included Caucasians, African Americans, Hispanics, and Asians. Inclusion criteria mandated that the Morphine Milligram Equivalent (MME) was 30–600 mg/day (males) and 20 to 180 mg/day (females) for treatment of chronic pain over 12 months. Ninety-six percent carried four or more risk alleles, and 73% carried seven or more risk alleles, suggesting a high predictive risk for opioid and alcohol dependence, respectively. These data indicate that chronic, legally prescribed opioid users attending a pain clinic possess high genetic risk for drug and alcohol addiction. Early identification of genetic risk, using the GARS test upon entry to treatment, may prevent iatrogenic induced opioid dependence.

## Introduction

Non-cancerous pain treatment is challenging for primary care medicine. The USA has faced an iatrogenically induced opiate/opioid epidemic that has killed thousands, with as many as 110 dying daily from a narcotic overdose [1, 2]. While some argue that big pharma was not the culprit, we fervently disagree with this retort. The driver in the surge in drug overdose mortality rates has been greater use of prescription opioid analgesics. Unintentional drug overdose deaths increased in 2007 to one every 19 minutes. Although initially more overdose deaths involved opioid analgesics than heroin and cocaine combined [3, 4], the recent availability of cheap street opiates has escalated heroin dependence [5–7]. By 2014, an NIH survey estimated that 25.3 million adults had pain every day for the preceding 3 months. In 2016–2017, many thousands of people died from opiate/opioid overdose, especially with the synthetic opioid fentanyl, which is more than 50 times more potent than other prescription opioids. To combat this growing threat to public safety, in 2016, new guidelines for prescribing opioids to chronic pain patients were issued by the Center for Disease Control (CDC) [6]. Morphine milligram equivalents declined by 29% in 2017, but more than 64,000 people still died from narcotic overdoses leading to a decrease in national life expectancy. Currently, nearly 116 million Americans suffer from chronic pain, according to the National Institute on Drug Abuse (NIDA). Those who suffer from severe pain are also likely to have worse overall physical and mental health status. Due to the role of big pharmaceutical industries in promoting opioid use and consequent addiction, the estimation is that they may have to pay $150 billion in fines.

**Fig. 1:**
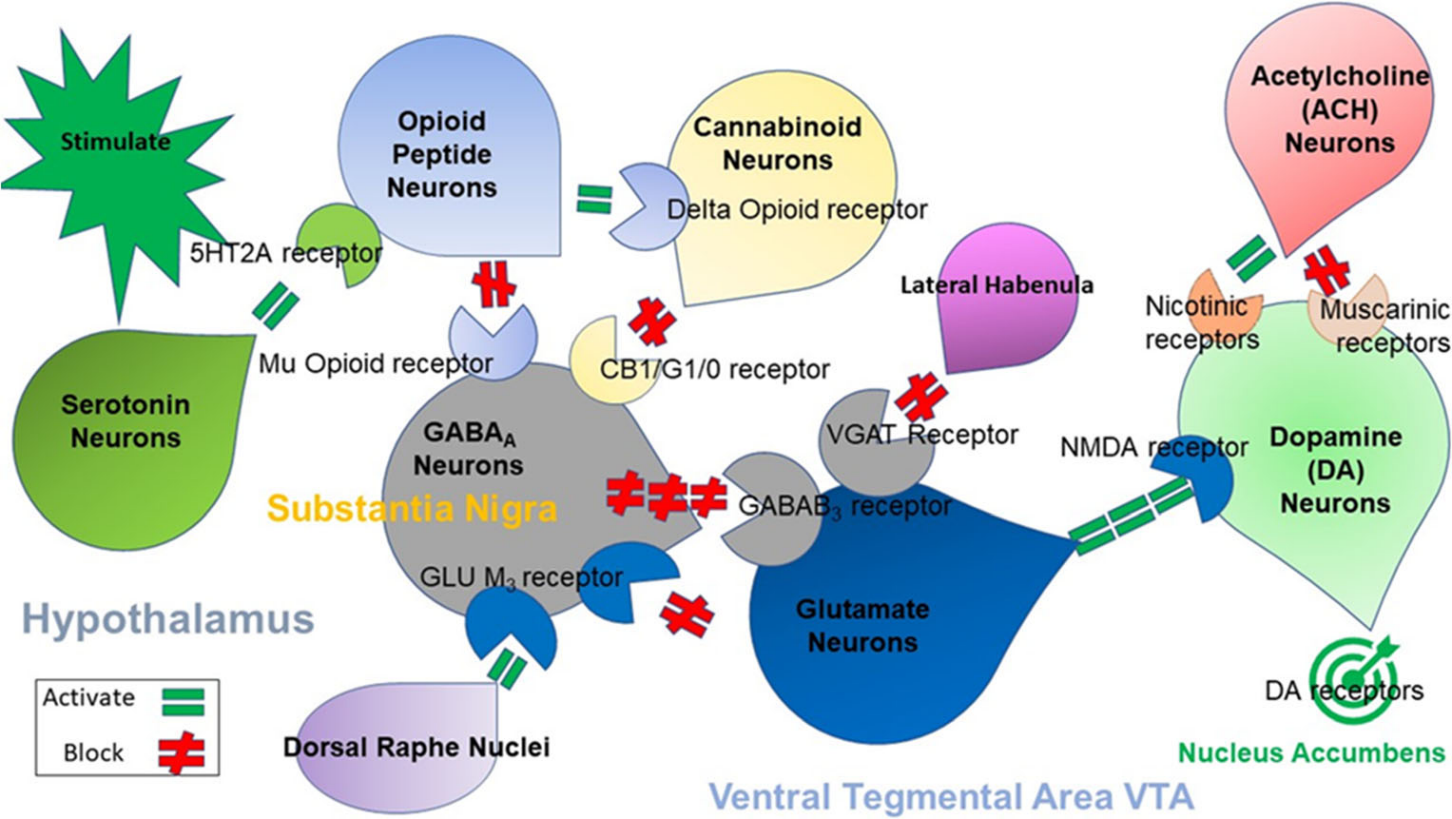
The brain reward cascade

The recommendation to mandate genetic testing before treating pain with potent synthetic opioids is an attempt to reduce this problem. The rationale for this recommendation requires understanding the neurochemical interactions of cannabinergic-endorphinergic-glutaminergic-cholinergic-dopaminergic systems (Fig. 1).

Figure 1 illustrates the interactions of 6 major neurotransmitter pathways within the brain reward cascade (BRC). Environmental stimulation in the hypothalamus causes serotonin release, which activates 5HT-2a receptors (green equal sign) that release opioid peptides from opioid peptide-containing hypothalamic neurons. These opioid peptides have two distinct effects acting, presumably, through two different opioid receptors: (1) they (possibly enkephalin) bind to mu-opioid receptors located on GABAergic neurons of the Substantia Nigra; (2) they (beta-endorphin) can bind to delta opioid receptors to stimulate cannabinoid neurons (anandamide and 2-archydonoglcerol), with consequent inhibition of GABA neurons at the substantia nigra. Cannabinoids, primarily 2-archydonoglcerol, can indirectly disinhibit GABAA neurons in the substantia nigra through G1/0 coupled activation to CB1 receptors. Similarly, glutamate neurons in the dorsal raphe nuclei (DRN) can disinhibit GABAA neurons in the substantia nigra through GLU M3 receptors’ activation. GABAA neurons can also inhibit VTA glutaminergic drive via GABAB3-containing neurons. Stimulation of ACH from cholinergic neurons in the nucleus accumbens can interact with both muscarinic and nicotinic receptors. Finally, VTA glutamate neurons that project to dopamine [DA] neurons can interact with NMDA receptors located on those neurons to cause dopamine release in the nucleus accumbens synaptic clefts.

Chronic opioid use results in clinical manifestations of dependency that include drug tolerance, hyper-analgesia, and potential addictive behaviors or reward deficiency syndrome (RDS) secondary to a hypodopaminergic state. However, there is a paucity of research related to the genetic risk or liability in chronic opioid users. A first step toward filling these holes was the investigation of the role of a reward gene panel, composed of ten genes and 11 risk-associated polymorphisms reflecting the brain reward circuitry, in 121 chronic opioid users attending several pain clinics in the USA. To accomplish this aim, we utilized RT-PCR for single nucleotide polymorphism (SNP) genotyping and multiplex PCR/capillary electrophoresis for fragment analysis, across dopamine receptors (DRD1-4), dopamine transporter (DAT1), GABA-B3 receptor (GABRB3), monoamine oxidase A (MAOA), mu opiate receptor (OPRM1), serotonin receptor (SLC6A4), and catecholamine-O-methyl-transferase (COMT).

### Pharmacogenetics

In terms of treatment efficacy and toxic consequences, each individual responds differently to each type of drug [7]. Factors that determine their responses include age, nutritional status, kidney and liver functions, concomitant illnesses, disease severity, and pathogenesis. Another factor is genetic variation (polymorphisms), which modifies the metabolism, efficacy, and toxicity of medications [8]. These inherited differences were first documented in the 1950s when genes that encode cholinesterase, the enzyme responsible for suxamethonium breakdown, unexpectedly caused prolonged muscle relaxation [9, 10]. The second gene-based drug response was documented when some patients treated with anti-malarial therapy carried a gene variant that lowered the activity of blood cell glucose 6-phosphate-dehydrogenase and bled to death [11]. These observed differences in drug response gave rise to the field of “pharmacogenetics.”

The genes that determine individual differences in drug response encode proteins. From 3 million unique DNA bases, these proteins form the molecular basis of cell cycle control and the synthesis or catabolism of structures like receptors, enzymes, and chemical messengers. These individual differences are due to gene variants of several genes involved in the multiple pathways of drug metabolism—individuals could carry gene variants that modify specific essential drug response-related proteins. The efficacy of medications will improve based on understanding specific molecular biological dysfunctions observable with accurate genotyping. Ongoing research and development that incorporates pharmacogenomics may lead to promising drug-based targets and make possible the design of effective novel medications.

Many molecular studies show that genetic polymorphisms modify sensitivity to specific medications. Notably, research concerning pharmacogenetic studies of opioid drugs is frequently reported and provides clinicians’ help regarding medication dosing [12]. Information regarding genetic polymorphisms and inter-patient variability in response to opioid therapy, the disposition (pharmacokinetics), and pharmacology (pharmacodynamics) is documented [13]. The pharmacogenetics related to opioids receptors, transporters, and enzymes include the cytochrome P450s, the ABC family of transporters, and opioid receptors, and uridine diphospho-glucuronosyl-transferases.

However, while pharmacogenetics has its place in pain management, little is known about the role played by known polymorphisms associated with reward genes and subsequent genetic addiction risk. This question provided the impetus to perform the present open pilot clinical trial in severe chronic opioid users attending pain clinics [14].

### Understanding GARS

The interaction of genes and neurotransmitters which control the release of dopamine is the brain reward cascade (BRC) [15] (see Fig. 1). Functional differences within the BRC, possibly genetic or epigenetic, may predispose individuals to addictive behaviors and altered pain tolerance [16]. The Genetic Addiction Risk (GARS) test is the first USA/European patented test clinically proven to predict vulnerability to pain and various other addictive and compulsive behaviors identified as reward deficiency syndrome (RDS).

Strategies to combat the opioid epidemic of prescription drug misuse and death and the implication of dopaminergic tone in pain pathways have been proposed previously [17]. The site of a predisposition to pain sensitivity may be the mesolimbic projection system, where genetic variations associate with pain vulnerability or tolerance [18]. These variations may provide specific targets to assist in the treatment of pain and identify risk for subsequent addiction. Many known gene variants are involved in, for example, opioid pharmacology, genetic testing of candidate genes like DRD1, 2, 3, 4, MOA-A, COMT, DAT1, SLC6A4, OPRM1, and GABRB3 might result in pharmacogenomics, personalized solutions, and improved clinical outcomes. Identifying those within compromised populations at genetic risk for RDS behaviors may be a frontline tool for better resource allocation in municipalities [19], especially in the criminal justice system.

The interaction of at least seven neurochemical pathways—serotonergic, GABAergic, endorphinergic, cannabinergic, glutaminergic, cholinergic, and dopaminergic—together constitute the “brain reward cascade” (see Fig. 1). This natural sequence of neurotransmission produces feelings of well-being. The cascade events, including the synthesis, vesicle storage, metabolism, release, and other neurotransmitter functions, are regulated by gene expression. Genetic testing of relevant variants can provide a window to an individual’s neurochemistry, assisting providers to formulate optimal treatment options.

The release of dopamine, the neurotransmitter responsible for motivation and stress reduction, is the neurological reward cascade’s functional endpoint. Consequently, genetically predisposed people with a hypodopaminergia seek out substances and behaviors to overcome this trait by activating mesolimbic brain dopaminergic centers [18, 20]. Lacking balanced dopamine function, an individual may have anhedonia, lack a sense of well-being, and may have difficulty with craving pleasure, lack of motivation, and coping with stress. Psychoactive substances and risky behaviors [21] induce DA release into the mesolimbic nucleus accumbens synapses to compensate for that individual’s hypo-dopaminergic trait/state.

Temporary relief from the discomfort and a sense of well-being is the product of this self-medication [22]. Pathological substance-seeking behaviors are employed to provide a pleasurable response and to decrease uncontrollable cravings. Chronic misuse of substances often leads to inactivation, downregulation, and inhibition of neurotransmitter synthesis and neurotransmitter depletion. Those individuals with risk-reward gene polymorphisms/variations, who experience environmental insults, will be at high risk for compulsive, impulsive, and addictive behaviors collectively referred to as reward deficiency syndrome (RDS), a spectrum that includes and characterizes genetically induced behaviors [23]. These pathological behaviors include addiction, tolerance, and dependence in chronic opioid use licit or illicit. The behavior or drug chosen by the individual is a function of both genetic and environmental factors such as availability of the drug and peer pressure.

**Fig. 2:**
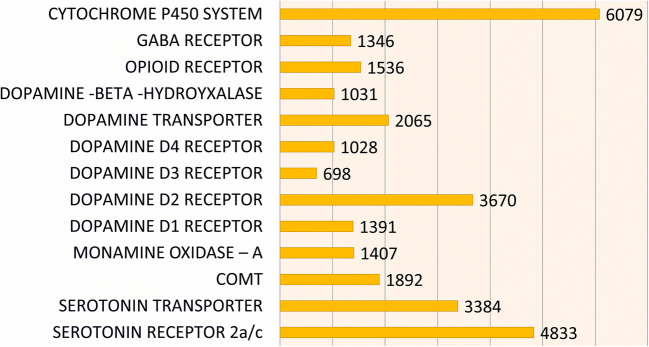
The number of studies published in PUBMED in 2014 for each risk allele selected for the GARS panel

Initially, 11 polymorphisms in ten genes selected for the development of genetic addiction risk scores (GARS) test are alleles that contributed most to the hypodopaminergic trait RDS and were chosen following an extensive literature review. The selection involved thousands of studies associated alleles with significant risk for addictive behaviors, both drug and non-drug RDS (see Fig. 2). In previous research from Blum et al.,[24] evaluating 273 mixed-gender patients attending seven treatment centers who completed the Addiction Severity Index (ASI-Media Version V), GARS significantly predicted drug severity (equal or >4 alleles) and alcohol severity (equal or >7 alleles). This previous research served as the basis for the present open pilot clinical trial.

Figure 2 is a graph of the number of studies for each risk allele selected for the GARS panel, published in PUBMED 2014, and used at the time to develop the GARS test.

## Materials and Methods

### Subjects and Demographics

In this study, we carefully selected severe but stable, chronic opioid-dependent patients (at least 12 months) derived from several pain clinics from San Antonio and Austin, Texas, New York, and Idaho in the USA. A total of 121 participants were identified and enrolled in the present clinical trial.

Inclusion criteria mandated that the overall pain score must be 6 out of 10. For the entire population, the average morphine equivalent (MME) must be 68 mg/d, with a range of 20–600 mg/day. The MME for males must be 102 mg/day, with a range of 30–600 mg/d. The MME of females must be 45 mg/d with a range of 20–180 mg/d, and each patient selected had been treated in each pain clinic for at least 12 months.

Of the 55 males (45%) and 66 females (55%), ethnicities were 67% Caucasian, Hispanic (17%), unknown classification (10%), African-Americans (4%), and Asian (2%). The average age for *M* = 54 years (range 15–88) and for *F* = 53 years (range 14–93) (see Table 1).

**Table 1:** Subject demographics

Population	All	Male	Female
Number (*n*)	121	55 (45%)	66 (55%)
Average age (*n*=121)	53	54	53
Ethnicity			
Caucasian	67%	36	45
Hispanic	17%	8	13
Unknown	10%	6	6
Black or African American	4%	4	1
Asian	2%	1	1

### Compliance with Ethical Standards

Study protocols were reviewed and approved by the University of Vermont, School of Medicine (Burlington, VT) and PATH Foundation (NY) Institutional Review Boards (IRB). For patient privacy protection, the genotyping data conformed to standard HIPAA and Genetic Information Non-Discrimination Act (GINA) practices mandated by law and de-identified. The participants provided and approved written informed consent.

### Sample Collection and Processing

Buccal cells were collected from each patient using an established minimally invasive collection kit, a sterile Copan 4N6FLOQ swab (Regular Size Tip In 109MM Long Dry Tube with Active Drying System). Subjects collected cells by rubbing the swab at least 25 times on both cheeks on each side of their mouth and then returned the swab to the specimen tube. Each respective pain clinic delivered the specimen tubes, labeled with a pre-defined bar-coded ID, to the Geneus Health Genomic Center for subsequent genotyping. Verified all steps of sample processing, used appropriate controls, including non-template controls and known DNA standards.

Tables 2, 3, 4, 5, and 6 index the genes and the specific risk polymorphisms included in the GARS panel. Each polymorphism was selected based on a known contribution to the RDS trait of hypodopaminergic functioning of the reward neurocircuitry. Capillary electrophoresis to detect AMELY and AMELX (AMELX’s intron 1 contains a 6 bp deletion relative to intron 1 of AMELY) and PCR amplification determined the sex of DNA samples.

**Table 2:** Single nucleotide polymorphisms (SNPs)

Gene	Polymorphism	Variant alleles	Risk alleles
Dopamine D1 receptor *DRD1*	rs4532	A/G	A
Dopamine D2 receptor *DRD2*	rs1800497	A/G (A1/A2)	A (A1)
Dopamine D3 receptor *DRD3*	rs6280	C/T	C
Dopamine D4 receptor *DRD4*	rs1800955	C/T	C
Catechol-O-methyltransferase *COMT*	rs4680	A/G (Met/Val)	G (Val)
Mu-opioid receptor *OPRMI*	rs1799971	A/G (Asn/Asp)	G (Asp)

**Table 3:** Simple sequence repeats (variable number tandem repeats and insertion/deletions)

Gene	Polymorphism	Variant alleles	Risk alleles
Dopamine D4 receptor *DRD4*	rs761010487	48bp repeat 2R-11R	≥ 7R, long form
Dopamine active transporter *DAT1*	rs28363170	40p repeat 3R-11R	<9R
Monoamine oxidase A *MAOA*	rs768062321	30bp repeat 2R-5R	3.5R, 4R, 5R
Serotonin transporter *SLC6A4 (5-HTTLPR)*	rs4795541, rs25531	43bp repeat, with SNP L/XL and S, G/A	S, LG

**Table 4:** Dinucleotide repeats

Gene	Polymorphism	Variant alleles	Risk alleles
GABA(A) receptor, Alpha-3 *GABRB3*	Rs764926719	CA dinucleotide repeat 171-201bp sized fragments	181

**Table 5:** GARS single nucleotide polymorphism assay information

Assay ID	Gene and SNP	Context sequence
C____1011777_10	*DRD1* rs4532	TCTGATGACCCCTATTCCCTGCTT [G/A] GGAACTTGAGGGGTGTCAGAGCCCC
C____7486676_10	*DRD2, ANKK1* rs1800497	CACAGCCATCCTCAAAGTGCTGGTC [A/G] AGGCAGGCGCCCAGCTGGACGTCCA
C_____949770_10	*DRD3* rs6280	GCCCCACAGGTGTAGTTCAGGTGGC [C/T] ACTCAGCTGGCTCAGAGATGCCATA
C____7470700_30	*DRD4* rs1800955	GGGCAGGGGAGCGGGCGTGGAGGG [C/T] GCGCACGAGGTCGAGGCGAGTCCGC
C___25746809_50	*COMT* rs4680	CCAGCGGATGGTGGATTTCGCTGGC [A/G] TGAAGGACAAGGTGTGCATGCCTGA
C___8950074_1_	*OPRM1* rs1799971	GGTCAACTTGTCCCACTTAGATGGC [A/G] ACCTGTCCGACCCATGCGGTCCGAA

**Table 6:** GARS repeats primer details

Primer	Sequence (5′ to 3′)	5′ Label	Reaction (nM)
AMELO-F AMEL0-R	CCC TGG GCT CTG TAA AGA ATA GTG ATC AGA GCT TAA ACT GGG AAG CTG	NED -	150
MAO-F MAO-R	ACA GCC TGA CCG TGG AGA AG GAA CGG ACG CTC CAT TCG GA	NED -	120
DAT-F DAT-R	TGT GGT GTA GGG AAC GGC CTG AG CTT CCT GGA GGT CAC GGC TCA AGG	6FAM -	120
DRD4-F DRD4-R	GCT CAT GCT GCT GCT CTA CTG GGC CTG CGG GTC TGC GGT GGA GTC TGG	VIC -	480
GABRA-F GABRA-R	CTC TTG TTC CTG TTG CTT TCA ATA CAC CAC TGT GCT AGT AGA TTC AGC TC	NED -	120
HTTLPR-F HTTLPR-R	ATG CCA GCA CCT AAC CCC TAA TGT GAG GGA CTG AGC TGG ACA ACC AC	PET -	120

### Biotechnical Development of GARS Test

Details about the biotechnical methods used to identify the (GARS) test alleles in Tables 2, 3, 4, 5, and 6 have been published previously [25, 26].

## Results

### Allele and Genotype Frequencies, GARS Severity Scores

**Fig. 3:**
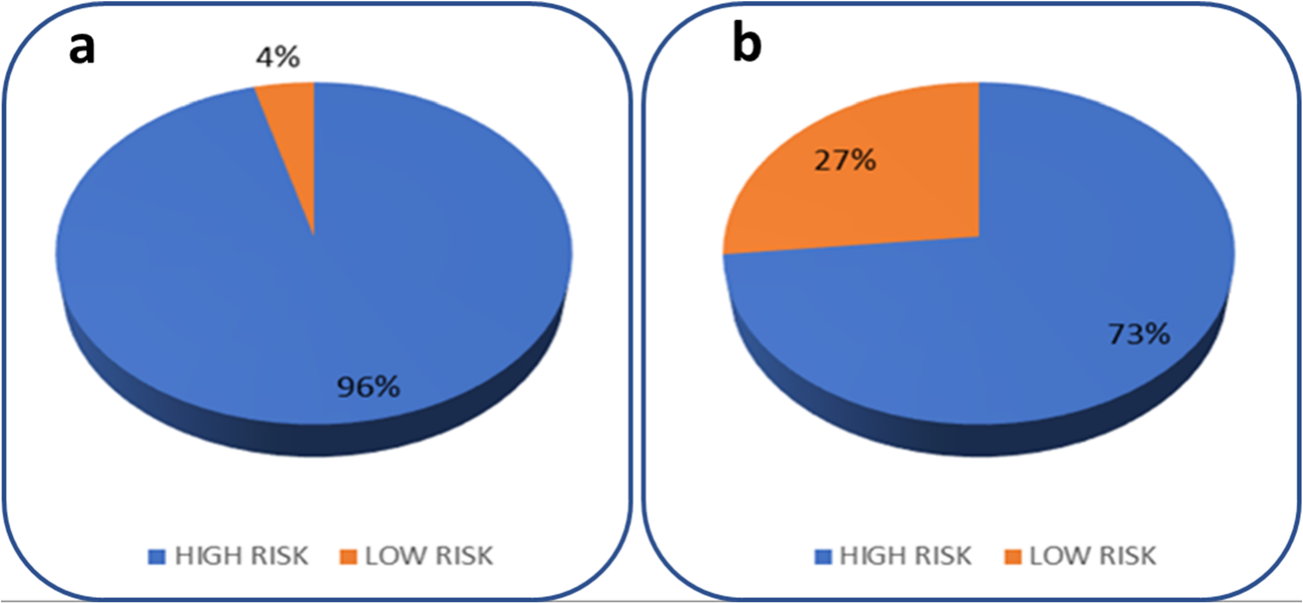
Percentage of 121 chronic pain patients predicted to carry elevated risk to drug (**a**) and alcohol (**b**) addiction based on the GARS genetic test

Figure 3a and b are pie charts of the GARS genotyping displaying the results for 121 subjects in the study. Figure 3 a illustrates the percentage of the 121 patients predicted to have an elevated risk for drug addiction (96%) by carrying at least 4 risk alleles; Figure 3b illustrates the percentage predicted to have an elevated risk for alcohol addiction (73%) by carrying at least 7 alleles.

In Fig. 3a, ninety-six (96%) percent of 121 patients carried at least four hypodopaminergia risk alleles, and in Fig. 3b, 73% carried at least seven. Previous research from Blum et al. [24], evaluating 273 mixed-gender patients attending seven various treatment centers who completed the Addiction Severity Index (ASI-Media Version V) GARS significantly predicted drug severity (equal or >4 alleles) and alcohol severity (equal or >7 alleles).

**Fig. 4:**
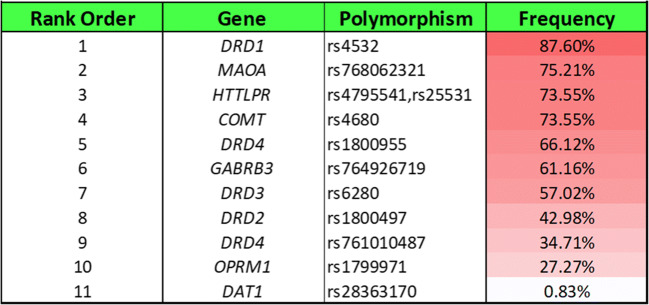
Ranking of GARS risk alleles by allele frequency

**Fig. 5:**
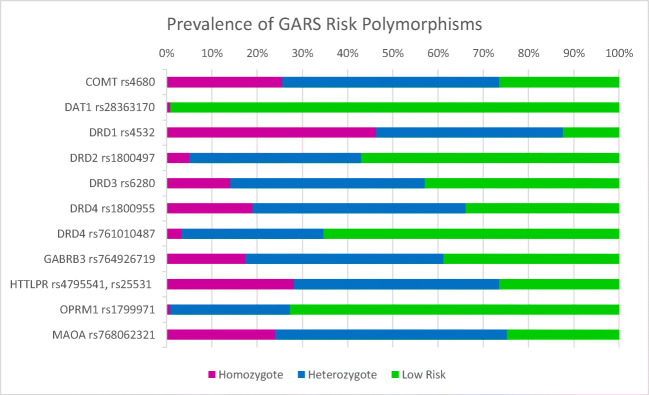
Prevalence of GARS risk polymorphisms for 121 chronic pain patients

Figure 4 reports the percentage of total calls (rank-ordered) that were risk alleles. While the DRD1 (rs 4532) at 88% ranked number 1 in terms of frequency, and the lowest risk allele was the DAT1 (rs 28363170) at 1%, we found the following rank order for the tested variants: DRD1 (rs 4532)> MAOA (rs 768O62321)> COMT (4680)> SLC6A4/ HTTLPR (rs 4795541, rs25531) > DRD4 (rs1800955)> GABRB3 (rs 7649267l9)> DRD3 (rs6280)> DRD2 (rs1800497)> DRD4 (rs 7610l0487)> OPRM1 (rs 1799971) > and DAT1 (rs 28363170). Figure 5 reports both heterozygous and homozygous analyses for each gene related to the prevalence of GARS risk. Refer to Table 2 for specifics about risk and non-risk variants.

Figures 4 and 5 represent the aggregate patient information for each gene in the GARS panel. Genotyping results include information about homozygosity and heterozygosity within the cohort.

## Discussion

The basis of the selection of the 121 patients was by the attending pain physician based on the mandated criteria detailed above as well as an additional criterion, namely, that the patient had to be stable with no positive urine screens for non-prescribed illicit drugs of abuse but not alcohol during urine screens. Indeed, a number of these individuals have shown periods of intoxication when attending the treatment center. The finding that 96% of these severe, chronic opioid-dependent patients showed a high GARS in this cohort may, at first glance, may be somewhat surprising, but it does support the hypothesis that concomitant chronic opioid users would have a high GARS score. It is somewhat surprising that the frequency of the OPRM1 allele occurred in these patients at the rate of 27.27% [27]. The belief that this finding could suggest that the real phenotype even in OUD (legal or possibly illicit) is not merely linked to the primary-opioid type of receptors but rather to generalized reward genes influencing overall dopamine release. If the general phenotype is confirmed to be hypodopaminergia rather than any individual allele (like OPRM1), then this will help pinpoint novel therapeutic targets. The US National Institute of Mental Health (NIMH) has introduced the Research Domain Criteria (RDoC) project [28] to overcome inadequacies of the DSM involved in focus on symptoms, and the division of psychiatric disorders, including pain issues, into distinct categories. The RDoC uses five brain systems as impaired domains in different psychiatric conditions. This alternative framework will influence neuroscience research to use current understanding of behavior–brain relationships as the starting point for clinical phenomenology.

There have been other studies by Blum et al. [29] showing the high prevalence of risk alleles such as the DRD2 Taq A1 in obesity and comorbid substance use disorder (SUD). Outpatient from Princeton, NJ, neuro-psychiatric clinic, were genotyped for presence or absence of the Taq A1 allele. They found the DRD2 A1allele present in 73.9% of the obese subjects with comorbid SUD and 23.5% of obese subjects with no SUD. While these studies do not relate directly to pain issues or genetics and epigenetics, they point out that addiction vulnerability to all addictive behaviors as subtypes of RDS is positively affected by genetic vulnerability independent of substance. For example, Blum’s group assessed substance use severity; they found that drug use increased with the Taq A1 allele prevalence. Of the less severe cases, 66.67% (8/12) possessed the A1 allele compared with 82% (9/11) of the most severe cases. Increasing drug use was positively and significantly associated with A1 allelic classification (*p* < 0.00001) in a linear trend analysis.

These data suggest that presence of the DRD2 A1 allele’s bolsters the risk for obesity and other related addictive behaviors (previously referred to as the reward deficiency syndrome). The study also confirms that a BMI over 25 by itself (without comorbid SUD) is not a sufficient criterion for association with the DRD2 A1 allele. An increase in addiction severity increases the prevalence of at least the DRD2 A1 allele, especially in Japanese alcoholics [30]. Arinami et al. [31] also found that the proportions of subjects with more severe alcoholism in the Japanese alcoholics (7 out of 7), 100% processed A1/A1, 62% (26 out of 42) had A1/A2, and 48% (10 out of 21) were least severe A2/A2. Importantly, when one considers the addiction risk severity issue as discussed earlier by Noble et al. [32] who reported that the number of brain DA D2 receptors was a function of genotype where A1/A1 had the highest reduction of DRD2 receptors compared with A1/A2 and A2/A2. These data support the concept that the DRD2 gene alone is associated with high addiction risk rates related to GARS selective risk alleles. Our finding in this cohort showed that the DRD2 A1 allele occurred in almost 1/3 of the population supports its continued importance even in legal chronic opioid users.

The finding of the highest frequency observed in this cohort, the DRD1 allele at 87.60%, is somewhat surprising, but if this holds up with a much larger OUD population, it may be a promising therapeutic target. Concerning the DRD1 allelic polymorphism as the most frequent allele, rs4532 studies by Liu et al. found evidence that SNPs related to DRD1 show an association with Chinese heroin dependence [33]. Other work by Peng et al. [34] found that single nucleotide polymorphisms (SNPs) of the DRD1 gene may be associated with the rapidity of the development of heroin dependence after the first opioid drug use. Of relevance to our present pilot study, the work of Zhu et al. [35] showing that for human carriers of the DRD1 rs4532, the duration of the transition from the first use to dependence (DTFUD), subjective pleasure responses to opioid on first use and post-dependence use and their opioid dependence overdose risk was significantly associated with the frequency of the DRD1 rs4532 allele. Our finding of a high (87.60.0 %) frequency of this allele in our present cohort is in complete agreement with Zhu et al. [35].

Additionally, Mayer-Blackwell et al. [36] found that oxycodone altered MAOA expression (found in this human study to have the second-highest frequency of 75.21%) in the dorsal striatum of high preferring alcohol C57BL/6J mice. However, there is a paucity of research involving high MAOA activity and chronic opioid use. With this stated, we have no explanation for the low presence of the DAT1 (dopamine transporter) rs28363170. Of interest, carriers of DAT1 rs28363170, as researched by Brewer et al. [37], showed that carriers of the 9-allele of the DAT1 3′-untranslated region [9,9 and 9,10] exhibited greater responses to cocaine for “high,” “any drug effect,” “anxious,” and “stimulated” (all *p*-values<0.001) compared with individuals homozygous for the 10-allele. However, more research is required in a larger cohort of pain patients presenting with chronic opioid use to determine if our finding of a low frequency of the DAT1 9-allele confers a four times greater DA synaptic reabsorption rate than the more common 10 allele.

This legal dilemma of the prescription of potent analgesics (like OxyContin®), possibly the main gateway to opioid addiction and abuse, could be prevented using the GARS test is to determine the risk for opioid dependence. Pain patients at risk of OUD could be treated with non-steroid analgesics and other forms of pain relief, such as electrotherapy, and avoid using opioid compounds.

Blum’s laboratory [38] proposed that any disturbance along the reward cascade (see Fig. 1), which might be due to either gene variations (polymorphisms) and environmental influences (epigenetics), can result in various addictive and other RDS behaviors. Despite the continued global search for specific candidate genes or clusters characterized by high-density SNP arrays and Genome-Wide Association Studies (GWAS), it is common knowledge that many attempts have failed to replicate or have been inconclusive. However, Palmer et al. [39] recently showed that between 25 and 36% of the genetic variance in the generalized vulnerability to substance dependence might be attributable to common rather than rare SNPs. Moreover, the effect of common SNPs is additive when shared across principal indicators of various comorbidities. As a result of such research studies [21–24, 27, 29–34, 40], growing evidence supports specific gene variants, which may account for risk-prediction.

Blum’s laboratory adopted a Bayesian approach [40] to establish that a positive predictive value (PPV) of 74%, specifically for the A1 variant of the D2 allele, appeared to be an indication that if a child is born with this polymorphism, they will have a much higher risk of future RDS behaviors at some point in their lives. Since the 1990 finding of the association of the TaqA1 allele of the DRD2 gene and severe alcoholism [38], laboratories across the globe, including NIDA and NIAAA, have confirmed this early work [21, 41] and extended the importance of various candidate genes and even second messengers in the reward system.

It is strategic to cautiously accept that obtaining better treatment results may stem from identifying reward circuitry gene polymorphisms linked to dopaminergic pathways and opioid receptors. Understanding the relationship between reward circuitry participation in chronic opioid outcomes and corresponding genotypes provides an innovative model to improve opioid replacement therapy and enhance a patient’s clinical experience [42], as suggested previously. Importantly, work from Gardner’s group [43] at NIDA showed that by using dopamine D3 receptor-knock-out (D3-KO) mice, low D3R availability in the brain represents a risk factor for the development of opioid abuse and addiction. This mouse data is in complete agreement with the findings of the present study.

Most importantly, while it is understood that opioids will have epigenetic effects on mRNA transcription and genetic expression of these risk alleles, our study only focuses on DNA polymorphisms. There are no alterations to these DNA polymorphisms, and thus the analysis of DNA can be done anytime during a person’s lifespan. The take-home message is that utilizing GARS upon entry into a pain clinic to predict risk for opioid-induced dependence vulnerability is preferable. While only 4% of this cohort did not display vulnerability to risk as identified via GARS testing and in all likelihood are dependent on opioids after a long-term (≥ 12 months), future studies would be most beneficial on people who do not carry the DNA polymorphic risks as a way of understanding potential protective mechanisms. Also, in a larger cohort, it may help explore racial and gender differences in risk for opioid dependence. Presently, there is significant evidence, for example, that specific genotypes such as A118G polymorphism of the OPRM1 gene caused different morphine consumption in female patients after total knee replacement [44–49].

## Summary

As shown in this cohort of pain patients, the GARS test represents a panel of the known brain reward genes and associated risk polymorphisms that bestow an increased genetic risk for addiction and other RDS behaviors and can be useful for medical monitoring and clinical outcome response measures [42]. The take-home message derived from this pilot open clinical trial is that these findings must be considered when deciding about drug prescription, especially to treat pain sensitivity, and the development of therapeutic approaches. More research to expand our results to other populations that may or may not meet DSM criteria for SUD is required [50, 51].
